# Assessing Outcomes of Patients Subject to Intensive Care to Facilitate Organ Donation: A Spanish Multicenter Prospective Study

**DOI:** 10.3389/ti.2024.12791

**Published:** 2024-04-12

**Authors:** Alicia Pérez-Blanco, María Acevedo, María Padilla, Aroa Gómez, Luis Zapata, María Barber, Adolfo Martínez, Verónica Calleja, María C. Rivero, Esperanza Fernández, Julio Velasco, Eva M. Flores, Brígida Quindós, Sergio T. Rodríguez, Beatriz Virgós, Juan C. Robles, Agustín C. Nebra, José Moya, Josep Trenado, Nieves García, Ana Vallejo, Eugenio Herrero, Álvaro García, Maria L. Rodríguez, Fernando García, Ramón Lara, Lucas Lage, Francisco J. Gil, Francisco J. Guerrero, Ángela Meilán, Nayade Del Prado, Cristina Fernández, Elisabeth Coll, Beatriz Domínguez-Gil

**Affiliations:** ^1^ Organización Nacional de Trasplantes, Madrid, Spain; ^2^ Hospital Universitario Puerta de Hierro, Madrid, Spain; ^3^ Hospital Universitario Vall d’Hebrón, Barcelona, Spain; ^4^ Hospital de la Santa Creu i Sant Pau, Barcelona, Spain; ^5^ Hospital Universitario de Navarra, Pamplona, Spain; ^6^ Hospital Universitario Ramón y Cajal, Madrid, Spain; ^7^ Hospital de San Pedro, Logroño, Spain; ^8^ Complejo Hospitalario Universitario, Santiago de Compostela, Spain; ^9^ Hospital Universitario Virgen del Rocio, Sevilla, Spain; ^10^ Hospital Universitario Son Espases, Palma de Mallorca, Spain; ^11^ Hospital Universitario La Paz, Madrid, Spain; ^12^ Hospital Universitario Central de Asturias, Oviedo, Spain; ^13^ Hospital Universitario Nuestra Señora de la Candelaria, Santa Cruz de Tenerife, Spain; ^14^ Hospital Clínico Universitario Lozano Blesa, Zaragoza, Spain; ^15^ Hospital Universitario Reina Sofía, Córdoba, Spain; ^16^ Hospital Universitario Miguel Servet, Zaragoza, Spain; ^17^ Hospital Universitario Virgen de la Arrixaca, Murcia, Spain; ^18^ Hospital Universitario Mútua Terrasa, Barcelona, Spain; ^19^ Hospital Universitario La Princesa, Madrid, Spain; ^20^ Hospital Universitario de Araba, Vitoria-Gasteiz, Spain; ^21^ Hospital Universitario de Torrevieja, Alicante, Spain; ^22^ Complejo Asistencial Universitario, Salamanca, Spain; ^23^ Complejo Hospitalario Universitario, Toledo, Spain; ^24^ Complejo Hospitalario Universitario, Albacete, Spain; ^25^ Hospital Universitario Virgen de las Nieves, Granada, Spain; ^26^ Hospital Álvaro Cunqueiro, Vigo, Spain; ^27^ Hospital General Universitario Santa Lucía, Cartagena, Spain; ^28^ Hospital Universitario de Torrecárdenas, Almería, Spain; ^29^ Fundación IMAS, Madrid, Spain; ^30^ Hospital Clínico Universitario de Santiago, Instituto de Investigaciones Sanitarias de Santiago, Santiago, Spain

**Keywords:** transplantation, deceased organ donation, death by neurologic criteria, devastating brain injury, intensive care to facilitate organ donation

## Abstract

Intensive Care to facilitate Organ Donation (ICOD) consists of the initiation or continuation of intensive care measures in patients with a devastating brain injury (DBI) in whom curative treatment is deemed futile and death by neurological criteria (DNC) is foreseen, to incorporate organ donation into their end-of-life plans. In this study we evaluate the outcomes of patients subject to ICOD and identify radiological and clinical factors associated with progression to DNC. In this first prospective multicenter study we tested by multivariate regression the association of clinical and radiological severity features with progression to DNC. Of the 194 patients, 144 (74.2%) patients fulfilled DNC after a median of 25 h (95% IQR: 17–44) from ICOD onset. Two patients (1%) shifted from ICOD to curative treatment, both were alive at discharge. Factors associated with progression to DNC included: age below 70 years, clinical score consistent with severe brain injury, instability, intracranial hemorrhage, midline shift ≥5 mm and certain types of brain herniation. Overall 151 (77.8%) patients progressed to organ donation. Based on these results, we conclude that ICOD is a beneficial and efficient practice that can contribute to the pool of deceased donors.

## Graphical Abstract

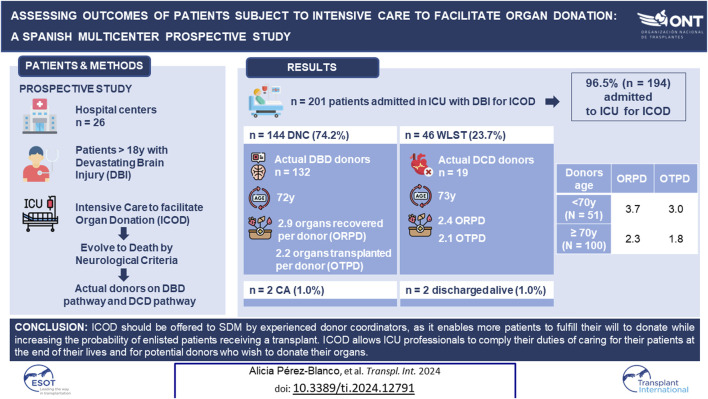

## Introduction

Intensive Care to facilitate Organ Donation (ICOD) is the initiation or continuation of intensive care measures in patients with a devastating brain injury (DBI) in whom curative treatment is deemed futile, and death by neurological criteria (DNC) is foreseen, with the aim of incorporating the option of organ donation into their end-of-life care plans.

ICOD is an established practice in Spain, with specific, published guidelines [1]. DBI is defined as a neurologic condition, assessed as an immediate threat to life or incompatible with good functional recovery, and where withholding or withdrawal of life-sustaining treatments (WLST) is being considered [2]. When a multidisciplinary treating team consensually decides to pursue end-of-life care, the patient is referred to the donor coordinator to evaluate donation opportunities and provide detailed information about ICOD to surrogate decision-makers (SDM). Having reflected upon the nuances of this donation process, the SDM may authorize ICOD to preserve the option of organ donation while awaiting DNC. Donor coordinators will inform the SDM that, if the patient does not meet DNC within the first 72 h or they revoke authorization, WLST will proceed.

ICOD in Spain contributes to 24%–33% of deceased donation activities, with a mean of 2.3 organs transplanted *per* donor [3–5]. Other countries—e.g. Australia [6], Canada [7], France [8], the Netherlands [9], the United Kingdom [10] and the United States [11]—have implemented similar policies, based on delaying WLST, to preserve the option of progressing to DNC. However, Spanish legislation and the ICOD national protocol permit the initiation of intensive measures, whilst several other national systems only accept their continuation [12–14].

Accurate prognosis of DBI early after the injury is difficult even for experienced clinicians, and a small percentage of patients with a DBI may be discharged alive with acceptable outcomes [10, 11, 15]. Additionally, ICOD requires the investment of expensive resources, with uncertainties about its effectiveness, and the possibility of unintended negative consequence on the patient, family and staff [3, 9, 10].

The aim of this study is to evaluate prospectively the outcomes of patients with a DBI admitted to the intensive care unit (ICU) for ICOD, and to identify clinical and radiological signs associated with progression to DNC. With an understanding of the most reliable signs, clinicians may identify and refer in a timely manner those patients most likely to become organ donors after DNC. A secondary objective is to measure the impact of ICOD on donation and transplantation metrics. The preliminary results of this research were published as an abstract in 2021 [16].

## Materials and Methods

**FIGURE 1:**
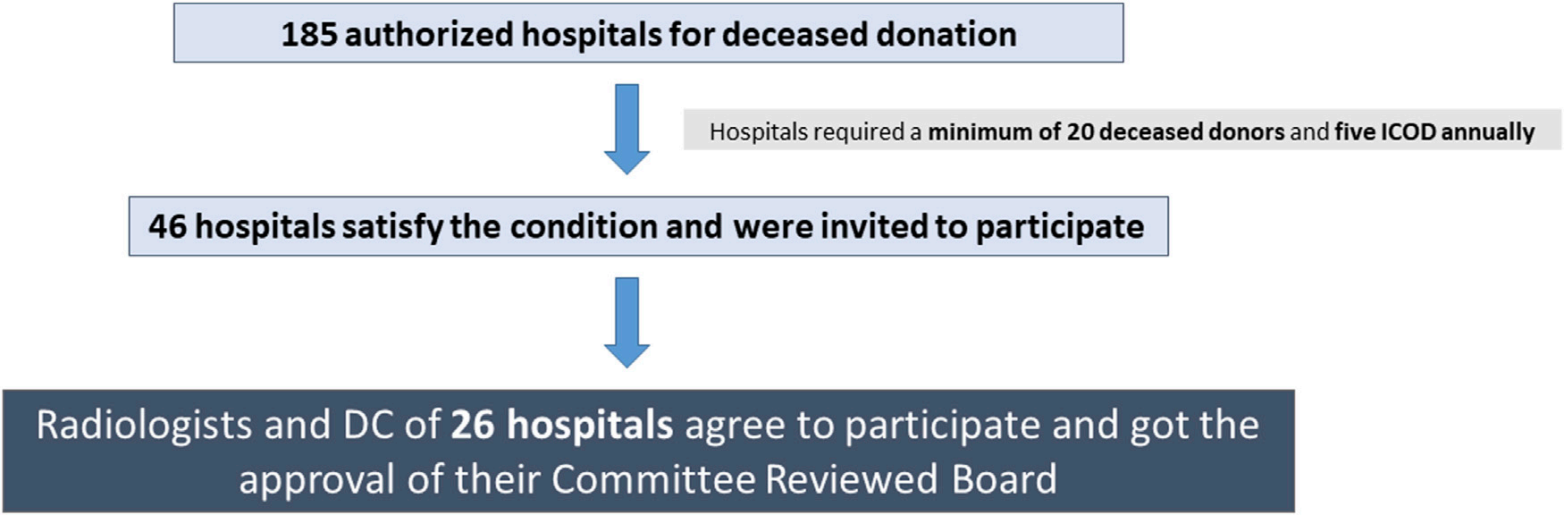
Hospital selection process to participate in the study. Footnote: DBD, Donation after Brain Death; DCD, Donation after Circulatory Death; DC, donor coordinator.

This is a prospective observational study conducted by the Organización Nacional de Trasplantes (ONT) and the Spanish Society of Intensive Care (SEMICYUC), performed in 26 Spanish hospitals (20 with neurosurgical units and 6 without) out of 46 hospitals invited to participate (Figure 1).

From July 2018 to July 2020, patients aged over 18 years, diagnosed with a DBI who had been admitted to the ICU for ICOD, were consecutively enrolled in the study.

Information was collected on patient demographics, location, and clinical and brain computed tomography (CT) scan data at the time of assessment for ICOD. Radiologists at participating centers completed a standardized form (Supplementary Material).

Information was also recorded on patients’ outcomes and transition to actual donors, where applicable.

For this study, severe brain damage (SBD) was defined based on values of validated scores for each etiology of the DBI: ICH score ≥3 for intracerebral hemorrhage score [17], HUNT-HESS ≥ IV for aneurysmal subarachnoid hemorrhage [18], NIHSS ≥25 for ischemic stroke [19] and GCS ≤5 for traumatic brain injury [20, 21]. An unstable patient was defined by the risk of imminent respiratory arrest [1].

Qualitative data is presented as absolute numbers and percentages. Quantitative data is displayed as mean and standard deviation or median and interquartile range (IQR), depending on the dispersion of the sample. Data derived from the clinical examination and brain CT at the time of assessment for ICOD, were evaluated for their potential association with progression to DNC [21–25].

Univariate effects were analyzed using Hazard Ratios (HR) and their 95% confidence intervals (95% CI). The analysis strategy is not only based on statistical criteria. Statistically significant variables (*p* < 0.05) identified on the univariate analysis, plus likely confounding variables, were included in the multivariate Cox model. Variance inflaction factor was used to study the collinearity between some explicative variables resulting after the univariate analysis. In case of collinearity, the variable with highest effect (HR) was considered the most appropriate to be included in the multivariate model. The assumption of proportionality of the models was evaluated. Discriminative ability was calculated by Harrell’s C index. Two-sided tests were used and a *p*-value < 0.05 was considered significant. Statistical analyses were performed using Stata version 17.0.

The study was approved by the Institutional Review Boards of each participating hospital. The Ethics Committee of ONT produced a written informed consent for SDMs enrolling in the study, which was endorsed by participating centers (Supplementary Material). All procedures were in accordance with the Declaration of Helsinki.

## Results

**FIGURE 2:**
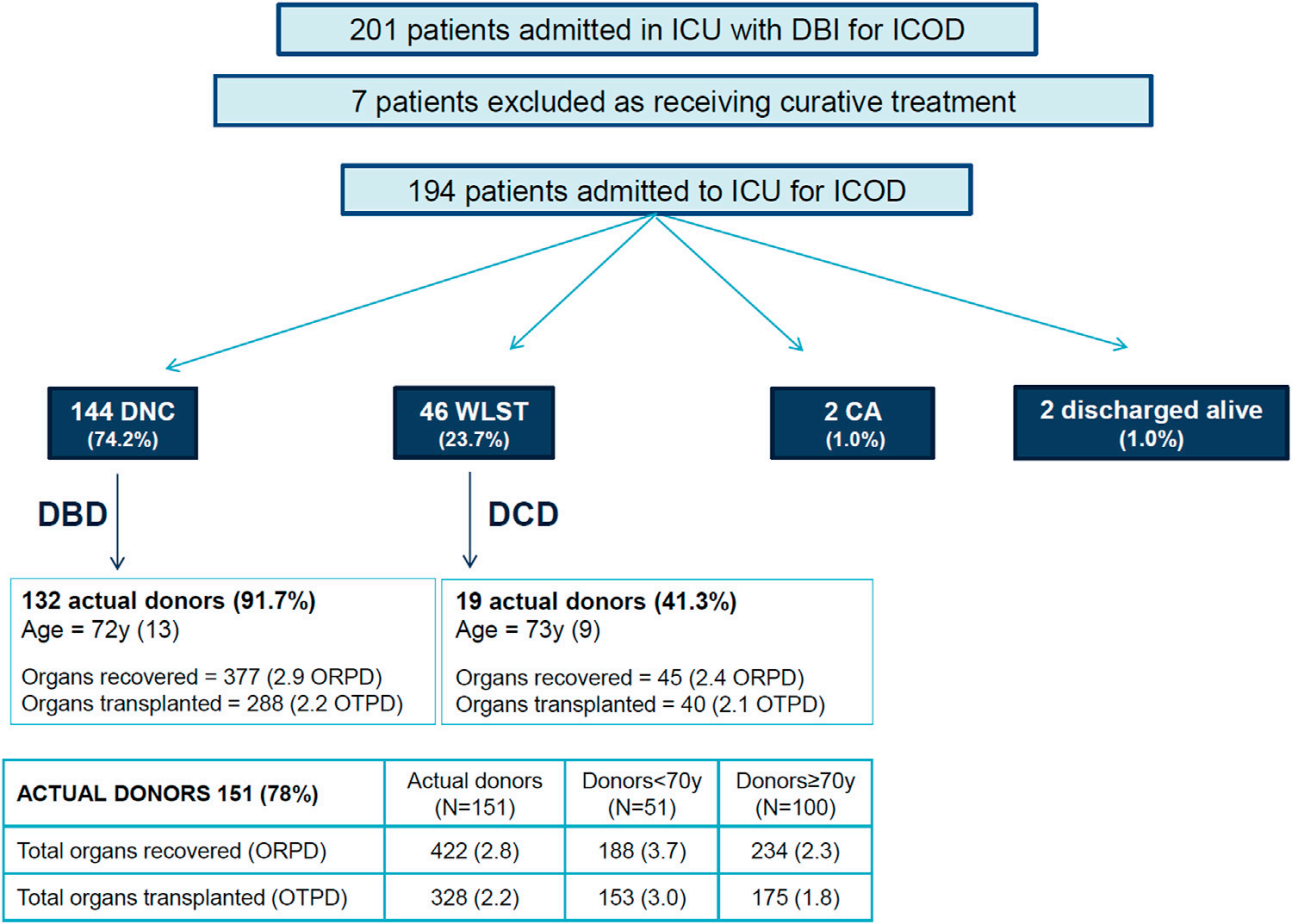
Evolution of patients subject to intensive care to facilitate organ donation and effectiveness on donation and transplantation. Footnote: DNC, Death by Neurologic Criteria; DBI, Devastating Brain Injury; WLST, Withdrawal of life sustaining treatment; CA, cardiac arrest; DBD, Donation after Brain Death; DCD, Donation after Circulatory Death; Age, mean (SD); ORPD, organ recovered per donor; OTPD, organ transplanted per donor.

In total, 201 patients with DBI were included in the study (Figure 2). Seven cases were excluded from analysis because patients had received medical treatment with curative intent within 24 h of the DBI. Data from the remaining 194 patients was analyzed.

Baseline characteristics of patients, location, results of the clinical examination and brain CT features at the time of ICOD assessment are shown in Table 1. The main cause of the DBI was an intracranial hemorrhage (n = 126, 88.1%). Assessment of the eligibility for ICOD was most frequently performed within the emergency department (n = 144, 74.2%). Most patients (n = 127, or 65.5%) were intubated and ventilated before the decision to apply ICOD. Brain CT showed 144 (74.2%) patients had a midline shift ≥5 mm, 150 (77.3%) had basal cistern effacement and 155 (79.9%) had some form of brain herniation.

**TABLE 1:** Characteristics of patients subject to intensive care to facilitate organ donation.

Demographic characteristics		
Sex male, n (%)	97	(50.0%)
Age (years), mean (SD)	72	(12)
Cause of DBI, n (%)		
Intracranial hemorrhage	126	(64.9%)
Traumatic brain injury	38	(19.6%)
Ischemic stroke/hypoxic brain injury	21	(10.8%)
Aneurysmal SAH	9	(4.6%)
Time and location where ICOD was assessed		
Time from DBI diagnosis to assessment for ICOD (hours), median (IQR)	1	(1–2)
Location of assessment for ICOD, n (%)		
Emergency room	144	(74.2%)
Intensive care unit	19	(9.8%)
Stroke unit	15	(7.7%)
Neurology ward	8	(4.1%)
Post-anesthesia care unit	4	(2.1%)
Other^a^	4	(2.1%)
Clinical data at the time of assessment for ICOD		
Intubated patient at the time of assessment for ICOD, n (%)	127	(65.5%)
Unstable (risk of imminent respiratory arrest), n (%)	17	(8.8%)
Glasgow Coma Score, n (%)		
3–5	155	(79.9%)
6–7	28	(14.4%)
≥8	11	(5.7%)
Severe brain damage^b^, n (%)	162	(83.5%)
ICH (*intracranial hemorrhage no-SAH, n = 126*) ≥ 3	111	(88.1%)
NIHSS (*ischemic CVA, n = 21*) ≥ 25	10	(47.6%)
HUNT-HESS (*aneurysmal SAH, n = 9*) ≥IV	8	(88.9%)
Glasgow Coma Scale (GCS) (*TBI, n = 38*) ≤ 5	33	(86.8%)
Radiological data at the time of assessment for ICOD		
Intracranial hemorrhage in temporal region, n (%)	51	(42.9%)
Midline shift (mm), median (IQR)	12	(4–16)
Midline shift ≥ 5 mm, n (%)	144	(74.2%)
ONSD^c^ 3 mm behind the globe (mm) (n = 104), mean (SD)	5.9	(1.3)
ONSD^c^ 10 mm behind the globe (mm) (n = 104), mean (SD)	5.1	(1.7)
Hydrocephalus, n (%)	104	(53.6%)
Basal cistern effacement, n (%)	150	(77.3%)
Herniation, n (%)	155	(79.9%)
Types of brain herniation, n (%)		
No herniation	39	(20.1%)
Transtentorial alone	54	(27.8%)
Subfalcine alone	50	(25.8%)
Cerebellar tonsil alone	7	(3.6%)
Transtentorial + Subfalcine	33	(17.0%)
Cerebellar tonsil + Transtentorial and/or Subfalcine	11	(5.7%)

^a^ Internal Medicine, Neurosurgery department, transfer from another hospital.

^b^ Severe Brain Damage is considered positive when any of the following occur: ICH≥ 3 for intracranial spontaneous hemorrhage; NIHSS ≥25 for ischemic stroke; HUNT-HESS ≥ IV, for aSAH; Glasgow ≤5 for TBI.

^c^ ONSD: optic nerve sheath diameter.

### Clinical Outcomes of Patients Subject to ICOD

Outcomes of patients subject to ICOD are displayed in Figure 2.

Of the 194 cases, 144 (74.2%) fulfilled the criteria for DNC after a median time of 25 h (95% IQR: 17–44) from ICOD onset, with most patients (n = 134, 69.1%) fulfilling DNC within the first 72 h from ICOD onset.

Forty-six patients (23.7%) died following the decision to WLST after a median time of 49 h (95% IQR: 24–84) from ICOD onset. The median time to death by circulatory criteria was 51 h (95% IQR: 25–84) after the initiation of ICOD. Two of the 46 patients were discharged alive from the ICU and transferred to the ward for palliative care, where they died (Table 2). In 21 patients, WLST took place within the first 48 h (11 due to medical contraindications and 10 because family revoked consent for ICOD). In the remaining 25 patients, WLST occurred after 48 h, in most cases because the timeframe agreed with SDM for DNC was surpassed.

**TABLE 2:** Characteristics of the four patients discharged alive from the intensive care unit.

Age	Etiology	GCS/Hunt-Hess	WLST (h)	Outcome
69	ICH	5	13	Palliative care, died in the ward
87	ICH	8	22	Palliative care, died in the ward
83	TBI	7		Discharged Alive; GOS 3
83	aSAH	>IV		Discharged Alive; GOS 3

aSAH, aneurysmal subarachnoid hemorrhage; ICH, intracranial hemorrhage; TBI, traumatic brain injury; GOS, 3: conscious, need help for daily tasks. WLST, withdrawal of life sustaining treatment in ICU.

Two patients (1.0%) admitted to the ICU for ICOD were later reassessed and received curative treatment. One of these patients had been diagnosed with an aneurysmal subarachnoid hemorrhage. The severity of the brain injury had been assessed close to the hemorrhage onset and SDM had refused any invasive therapeutic intervention. However, after being reassessed in the ICU, their neurological condition showed improvement, and the decision was made to apply curative treatment. After 26 days, the patient did not show any neurological improvement and was transferred to internal medicine. The second patient had been diagnosed with a traumatic brain injury and transferred from another hospital for ICOD. Clinicians reassessed the neurological status and recommended shift to curative treatment, despite the severity of the brain injury. After 15 days their neurological condition did not improve and they were discharged to a social institution.

### Factors Associated With Progression to DNC

Univariate and multivariate analyses of factors associated with progression to DNC in ICOD patients is shown in Table 3. The variables Glasgow Coma Score and intubated patient were not included in the final multivariable model due to their collinearity with severe brain damage and unstable respectively. On the final multivariate Cox model, multiple factors were significantly associated with progression to DNC including: age under 70 years, severe brain damage, instability at the time of assessment for ICOD, intracranial hemorrhage in the temporal region, midline shift ≥5 mm and certain types of brain herniation (cerebellar tonsillar herniation combined with transtentorial and/or subfalcine herniation).

**TABLE 3:** Analysis of the factors associated with death by neurological criteria in patients subject to intensive care to facilitate organ donation. Univariate and multivariate Cox model.

Variables	Univariate			Multivariate^a^		
	Hazard ratio	[CI 95% HR]	*p*	Hazard ratio	[CI 95% HR]	*p*
**Sex male**	1.06	[0.76–1.47]	0.737			
**Age <70 years^b^**	**1.74**	**[1.24**–**2.45]**	**0.002**	**1.78**	**[1.24–2.56]**	**0.002**
**Cause of death**			0.301			
*Aneurysmal subarachnoid haemorrhage*	***Ref.***					
Hemorrhagic Stroke	1.59	[0.70–3.64]	0.270			
Ischemic Stroke/Hypoxic brain injury	1.00	[0.39–2.62]	0.994			
Traumatic brain injury	1.36	[0.56–3.32]	0.497			
**Glasgow Come Score**			0.058			
** 3–5**	**3.31**	**[1.22–8.98]**	**0.019**			
6–7	2.88	[0.98–8.49]	0.055			
*≥8*	***Ref.***					
**Severe Brain Damage^c^**	**1.87**	**[1.11–3.14]**	**0.019**	**2.06**	**[1.19–3.58]**	**0.010**
Time from DBI diagnosis to ICOD evaluation	0.95	[0.88–1.02]	0.131			
**Intubated patient**	**1.57**	**[1.10–2.25]**	**0.014**			
Unstable (risk of imminent respiratory arrest)	**1.73**	**[0.98–3.07]**	**0.059**	**3.29**	**[1.71–6.33]**	**<0.001**
Intracranial hemorrhage	1.37	[0.96–1.95]	0.079			
**Intracranial hemorrhage in temporal region**	**1.70**	**[1.21–2.39]**	**0.002**	**1.47**	**[1.03–2.10]**	**0.034**
**Midline shift ≥5 mm**	**1.68**	**[1.13–2.51]**	**0.010**	**1.77**	**[1.14–2.74]**	**0.011**
ONSD^d^ 3 mm behind the globe	0.91	[0.76–1.08]	0.265			
Hydrocephalus	0.92	[0.67–1.28]	0.639			
Basal cistern effacement	1.36	[0.90–2.05]	0.146			
**Type of brain herniation**			**0.003**		***Ref***	
*No herniation*	***Ref.***					
** Transtentorial**	**1.98**	**[1.18–3.32]**	**0.009**			
Subfalcine	1.41	[0.83–2.40]	0.204			
** Cerebellar tonsil**	**2.55**	**[1.03–6.34]**	**0.043**			
Transtentorial + Subfalcine	1.63	[0.92–2.89]	0.097			
**Cerebellar tonsil + Transt. And/or Subfalcine**	**4.73**	**[2.15–10.40]**	**<0.001**	**1.45**	**[0.99–2.12]**	**0.054**

^a^ Discriminate analysis: Harrell Index, C 0.66.

^b^ Cut-off stablished through ROC, curve.

^c^ Severe Brain Damage, defined by an ICH ≥ 3 for intracranial spontaneous hemorrhage, an NIHSS ≥25 for ischemic stroke, a HUNT-HESS ≥ IV, for aneurysmal subarachnoid haemorrhage and a Glasgow Coma Score ≤5 for traumatic brain injury.

^d^ ONSD: Optic Nerve Sheath Diameter.

Bold means statistically significant, defined as *p* equal to or less than 0.05.

### Impact of ICOD on Organ Donation and Transplantation

Overall, 151 (77.8%) patients transitioned to actual organ donors, 132 after DNC and 19 after the circulatory determination of death (41.3% of the 46 patients who died after the WLST). In total, 2.8 organs were recovered and 2.2 organs were transplanted *per* actual donor (1.8 for donors aged ≥70 years) (Figure 2).

The reasons why patients with DNC did not transition to actual donation were: medical contraindications (n = 6), SDM refused consent (n = 4), no suitable recipient (n = 1) and unexpected cardiac arrest after DNC (n = 1). Corresponding reasons why patients who died after the WLST did not transition to actual donation were: medical contraindications (n = 13), age unsuitable for donation after the circulatory determination of death (DCD) (n = 12) and death not expected within a timeframe suitable for organ donation (n = 2).

## Discussion

Even for experts in neurocritical care, prognostication in DBI within a short timeframe from injury is challenging. With this study, we wanted to evaluate the practice of ICOD and provide detailed patient outcomes. To the best of our knowledge, this is the first multicenter study that prospectively evaluates the impact of clinical and radiological data from patients with DBI and upon the likelihood of progression to DNC in patients admitted in ICU for ICOD.

### Factors Associated With Progression to DNC

Most patients subject to ICOD in our series did progress to DNC (74.2%), consistent with reports from retrospective multicenter and single-center studies performed in Spain [3–5]. This progression to DNC was higher than that reported by both Melville et al. (65%) and Humbertjean et al. (23%) [6, 25]. Differences may be due to variation between patient cohorts and also improving ability to prognosticate over time.

Clinical factors independently associated with the progression to DNC were: age under 70 years, achieving SBD criteria (diagnosis specific), and risk of imminent respiratory arrest. Relevant radiographic factors consisted of intracranial hemorrhage in the temporal region, midline shift ≥5 mm and a combination of tonsil with transtentorial and/or subfalcine herniation.

Being older than 70 has been well described as a factor reducing the likelihood of progression to DNC [3–5, 25, 26]. This finding should not prevent clinicians considering ICOD in older patients, as our study included 100 donors aged ≥70, resulting in 1.8 organs transplanted *per* donor (Figure 2).

Although some have reported a cut-off value in GCS (e.g., GCS≤6) to be associated with progression to DNC [21], others do not identify a firm cut-off value [10, 15]. Aware of this limitation, we identified positive indicators for defining Severe Brain Damage (SBD) depending on the brain injury pathology (ICH≥ 3 for intracranial spontaneous hemorrhage, NIHSS ≥25 for ischemic stroke, HUNT-HESS ≥ IV for aneurysmal subarachnoid hemorrhage and a Glasgow Coma Scale ≤5 for traumatic brain injury). Our results show that meeting criteria for SBD is associated with progression to DNC in this selected cohort of ICOD patients (OR 2.06 [1.19–3.58]).

We evaluated the findings of the brain CT scan performed when ICOD was considered, to assess their association with progression to DNC. This approach is different from that of Ray A et al. who analyzed signs in the CT scan taken before DNC occurred [27]. We like others found herniation is associated with DNC [21, 25]. The combination of tonsillar plus transtentorial and/or subfalcine herniation was the combination most strongly associated with progression to DNC in patients subject to ICOD. This contrasts with Ray et al, who did not observe any association between herniation and DNC [27]. This may be due to the different timing of the brain CT to DNC and the highly selective cohort of patients in our study [21, 25, 27].

### Clinical Outcomes of Patients Subject to ICOD

ICU admission for ICOD allows stabilizing hemodynamic and respiratory parameters, reassessing the neurological condition, and studying thoroughly the patient’s medical history to establish eligibility for organ donation. The neurological reassessment of patients with DBI is performed daily to evaluate clinical improvement or deterioration and, when needed, a brain CT-scan is repeated.

The median time to meet DNC in our study (25 h) was relatively short compared to the average of 43 h from ICU admission (IQR 24–87) observed in all DBI patients who ultimately progressed to DNC. This latter cohort includes 2,393 patients admitted to ICU (26 centres) with DBI for either treatment with curative intent or ICOD from 2018 to 2020. The implemented ICOD practice in Spain shows that donor coordinators are highly restrictive and only consider admittance in ICU for ICOD a patient with DBI that will otherwise be admitted for terminal sedation, which explains the advanced age of our sample.

Two patients (1%) in our cohort were discharged alive (both aged 83), similar to 0.9% reported by Melville et al [6]. In both cases, the treating team decided to shift from ICOD to full treatment after observing an improvement in the neurological exam.

The main reason for not transitioning to DNC was WLST (n = 46). Reasons for the WLST in the first 48 h from ICOD onset were medical contraindications (N = 11) and SDM revoking consent for ICOD before the end of the agreed-upon timeframe (N = 10). After 48 h, WLST occurred in most cases because the timeframe agreed with SDM for DNC was surpassed (N = 23) or SDM revoked consent before surpassing it (N = 2).

The majority of the 11 medical contraindications arose as a result of serological and radiographic tests after ICU admission. We must reinforce the importance of learning from the relatives about the donor’s habits, in order to perform tests before admission to ICU for ICOD.

### Impact of ICOD on Organ Donation and Transplantation

ICOD requires investment of human and financial resources. Our study helps to confirm it is an efficient policy, considering 78% of patients subject to ICOD transitioned to actual donors, with a rate of 2.2 organs transplanted per donor. The percentage of ICOD patients transitioning to actual donation is lower in the series published by Melville et al. (52%) and Witjes et al (42%) [6, 9]. However, we included all cases with consent for ICOD, while theirs additionally recorded cases with declined consent for admission to the ICU to enable organ donation.

Several authors gauge the financial savings in hemodialysis, as well as the recipients’ quality-adjusted life-years gained, and compare these figures to a relatively short stay in the ICU of patients admitted for ICOD. They also conclude that implementing an ICOD protocol or a ‘DBI pathway’ is highly efficient from the transplantation perspective [10, 26–28].

Some have questioned the additional stress placed on families agreeing to ICOD [29, 30]. Conversely, others found that long admissions of older patients with cerebrovascular injuries help their relatives grasp the reality of their loss, with a positive correlation with organ donation [31]. Many emphasized the crucial role of a positive environment around donation in the ICU, highlighting fluent communication between clinicians and donor coordinators as a means to support families’ decision making [32, 33].

Twelve families expressed fatigue around the ICU admission, requesting WLST before the end of the agreed-upon timeframe. However, there were no refusals of DCD donation in the WLST group, so the SDMs’ initial decision to authorize organ donation persisted, but prolongation of waiting in ICU for DNC was not felt to be tolerable to the SDM.

### ICOD Protocols Throughout the World

The efficacy of the Spanish ICOD protocol may be explained by its differences from those in other countries. First, while in Netherlands emergency care physicians approach families in the emergency department to propose ICU admission to preserve organ donation, in Spain, the donor coordinator leads the process, informing SDM about ICOD once the decision has been made not to proceed with a therapeutic purpose [9]. The special training of donor coordinators in approaching families has shown to improve the likelihood of consent to organ donation [3].

Second, though most patients in our study had been diagnosed with a DBI in the emergency room, cases were also identified in other hospital units, producing 26% of the candidates for ICOD.

A third important difference lies in the advanced age of patients subject to ICOD in our study, compared with other published articles [6, 8–11]. The mean age of ICOD patients in our study (72 years), contrasts with the Australian mean age of patients included in the “potential organ donation pathway” (55 years) [6] and with the mean age of patients admitted to the ICU for organ donation in Netherlands (59 years) [9] or in France (66 years) [25]. The advanced age of patients subject to ICOD in our series is in accordance with previous studies [3–5] and the established national policy on utilizing organs obtained from expanded-criteria donors [28].

### Strengths and Limitations of This Study

This is the first prospective study in the field that may shed light on the impact of a nationwide ICOD policy on patient outcomes, and donation and transplantation metrics.

Limitations are related to sample size, resources and availability of specialized clinicians between hospitals with and without neurosurgical departments. Indeed, premature neurological assessment of the patient with the aim of transporting them to a neurosurgical center may be misleading.

The interobserver variability inherent in a multicenter study affects the interpretation of both the clinical and radiological results.

Another limitation is associated with the early performance of the brain CT. Some radiographic signs of intracranial hypertension may not be visible at the time of this exam, impeding the radiologists’ ability to clearly observe the signs of impending progression to DNC. Yet, in medical practice, evaluation of a patient with DBI as an ICOD candidate is based on the results of the radiology performed during diagnosis of DBI.

### Conclusion

Clinical and radiographic factors identified in our study may help clinicians identify patients potentially progressing to DNC, permitting efficient utilization of ICU resources and an effective approach to families.

ICOD should be offered to SDM by experienced donor coordinators, as it enables more patients to fulfill their will to donate while increasing the probability of enlisted patients receiving a transplant.

Our findings reinforce the importance of providing information to SDM about all the uncertainties involved in this complex process, so they can envision the potential obstacles for their loved ones to become a donor after DNC, and make a fully-informed decisions around consent. Intensivists and donor coordinators should have a plan to proceed with WLST in cases of medical contraindication, at the request of family, or if the patient does not progress to DNC by a pre-agreed timeframe.

Future large prospective studies are required to further validate and build upon these important results that may ultimately increase the number of organs available for donation.

## Data Availability

The original contributions presented in the study are included in the article/Supplementary Material, further inquiries can be directed to the corresponding author.

## Abbreviations

CI, confidence intervals; CT, computed tomography; DBI, devastating brain injury; DNC, death by neurological criteria; GCS, Glasgow Coma Scale; HR, Hazard Ratios, ICH, Intracerebral Hemorrhage Score; ICOD, Intensive Care to facilitate Organ Donation; ICU, Intensive Care Unit; IQR, interquartile range; NIHSS, National Institutes of Health Stroke Scale; ONT, Organización Nacional de Trasplantes; SBD, severe brain damage; SDM, surrogate decision-makers; SEMICYUC, Spanish Society of Intensive Care and Coronary Units; WLST, withdrawal of life-sustaining treatments.
