# Supplementary material for: Assessing Outcomes of Patients Subject to Intensive Care to Facilitate Organ Donation: A Spanish Multicenter Prospective Study
Source: Transpl Int. 2024 Apr 12;37:12791. doi: 10.3389/ti.2024.12791 (PMC11046399; doi:10.3389/ti.2024.12791)
Supplement: Supplementary file 1 [file DataSheet2.pdf]

INSTITUTIONAL REVIEW BOARDS

STUDY TITTLE

"**Evolución final de los pacientes con daño cerebral catastrófico que ingresan en los Servicios de Medicina Intensiva de los hospitales del Sistema Nacional de Salud como posibles donantes para Cuidados Intensivos destinados a la Donación de Órganos**"

Final evolution of patients with catastrophic brain damage who are admitted to the Intensive Care Units of the hospitals of the National Health System as possible donors for Intensive Care to facilitate Organ Donation"

**BOARD NAME**	Hospital Universitario La Paz Madrid		
**APPROVAL Nº**	PI-3399	**APPROVAL DATE**	08/08/2018
**BOARD NAME**	Hospital Universitario Vall D´Hebrón Barcelona		
**APPROVAL Nº**	PR(AMI)358/2018	**APPROVAL DATE**	11/09/2018
**BOARD NAME**	Hospital Universitario Virgen De La Arrixaca Murcia		
**APPROVAL Nº**	2018-9-5-HCUVA	**APPROVAL DATE**	24/09/2018
**BOARD NAME**	Hospital Universitario Puerta De Hierro Madrid		
**APPROVAL Nº**	HUPH-CEIC-13-18	**APPROVAL DATE**	09/07/2018
**BOARD NAME**	Hospital Universitario De La Santa Creu i Sant Pau Barcelona		
**APPROVAL Nº**	HSCSP18/297	**APPROVAL DATE**	16/07/2018
**BOARD NAME**	Complejo Hospitalario Universitario De Canarias. Santa Cruz De Tenerife		
**APPROVAL Nº**	CHUNSC_2018_39	**APPROVAL DATE**	13/09/2018
**BOARD NAME**	Hospital Universitario Ramón y Cajal Madrid		
**APPROVAL Nº**	HURYC-357	**APPROVAL DATE**	12/02/2019
**BOARD NAME**	Hospital Universitario Central De Asturias Oviedo		
**APPROVAL Nº**	HUCA-CEIC- 209/18	**APPROVAL DATE**	14/08/2018

**ONT ETHICS COMMITTEE BOARD**			
**APPROVAL Nº**	2018/CIOD-02	**APPROVAL DATE**	06/06/2018

The ethics committees detailed below approved the study based on the ONT ethics committee and the abovementioned centers positive evaluations, endorsing the informed consent produced by ONT ethics committee.

**BOARD NAME**	Complejo Hospitalario Universitario De Vigo		
		**APPROVAL DATE**	15/10/2018
**BOARD NAME**	Hospital Universitario de Navarra Pamplona		
		**APPROVAL DATE**	25/07/2018
**BOARD NAME**	Hospital Universitario de San Pedro Logroño		
		**APPROVAL DATE**	06/08/2018
**BOARD NAME**	Complejo Hospitalario Universitario Santiago de Compostela		

		**APPROVAL DATE**	02/08/2018
**BOARD NAME**	Hospital Universitario Virgen del Rocío Sevilla		
		**APPROVAL DATE**	17/07/2018
**INSTITUTIONAL REVIEW BOARDS**			
**BOARD NAME**	Hospital Universitario Son Espases, Palma de Mallorca		
		**APPROVAL DATE**	02/08/2018
**BOARD NAME**			
		**APPROVAL DATE**	18/08/2018
**BOARD NAME**	Hospital Universitario Reina Sofía Córdoba		
		**APPROVAL DATE**	24/08/2018
**BOARD NAME**	Hospital Universitario Miguel Servet Zaragoza		
		**APPROVAL DATE**	31/08/2018
**BOARD NAME**	Hospital Universitario Mútua Terrasa Barcelona		
		**APPROVAL DATE**	13/08/2018
**BOARD NAME**	Hospital Universitario La Princesa Madrid		
		**APPROVAL DATE**	06/09/2018
**BOARD NAME**	Hospital Universitario de Araba Vitoria-Gasteiz		
		**APPROVAL DATE**	06/09/2018
**BOARD NAME**	Hospital Universitario de Torrevieja Alicante		
		**APPROVAL DATE**	13/09/2018
**BOARD NAME**	Complejo Asistencial Universitario Salamanca		
		**APPROVAL DATE**	13/09/2018
**BOARD NAME**	Complejo Hospitalario Universitario Toledo		
		**APPROVAL DATE**	12/09/2018
**BOARD NAME**	Complejo Hospitalario Universitario Albacete		
		**APPROVAL DATE**	05/09/2018
**BOARD NAME**	Hospital Universitario Virgen de las Nieves Granada		
		**APPROVAL DATE**	04/10/2018
**BOARD NAME**	Hospital General Universitario Santa Lucía Cartagena		
		**APPROVAL DATE**	09/10/2018
**BOARD NAME**	Hospital Universitario de Torrecárdenas Almería		
		**APPROVAL DATE**	15/10/2018
