# Supplementary material for: Assessing Outcomes of Patients Subject to Intensive Care to Facilitate Organ Donation: A Spanish Multicenter Prospective Study
Source: Transpl Int. 2024 Apr 12;37:12791. doi: 10.3389/ti.2024.12791 (PMC11046399; doi:10.3389/ti.2024.12791)
Supplement: Supplementary file 2 [file DataSheet1.pdf]

Brain CT-scan at the time of Intensive Care to facilitate Organ Donation assessment

Identification

CIOD CODE:___________

Etiology catastrophic brain damage HEAD CT SCAN

1. CEREBRAL HEMORRHAGE: YES/NO *(If yes, complete the following)*

1.2 Location *(multiple answers adimtted):*

- ○ Frontal
- ○ Parietal
- ○ Occipital
- ○ Temporal
- ○ Basal Ganglia
- ○ Brain Stem

1.3 Volume: ____cc

1.4 Extension to ventricles: YES/NO

1.5 Basal cistern effacement: YES/NO

1.6 Midline shift: ______mm

1.7 Herniation: YES/NO

Type:
- ○ transtentorial
- ○ subfalcine
- ○ Cerebellar tonsil

1.8 Hydrocephalus: YES/NO

1.9 Fisher Scale (if SAH):

1.10 Optic Nerve Sheath Diameter

3 mm behind the globe:____
10 mm behind the globe:____

2. DIFFUSE BRAIN ISCHEMIA: YES/NO *(If yes, complete the following)*

2.1 Location *(multiple answers adimtted):*

- ○ Frontal
- ○ Parietal
- ○ Occipital
- ○ Temporal
- ○ Basal ganglia
- ○ Brian stem

2.2 Basal cistern effacement: YES/NO

2.3 Midline shift ______mm

2.4 Herniation: YES/NO

Type:
- ○ transtentorial
- ○ subfalcine
- ○ Cerebellar tonsil

2.5 Hydrocephalus: YES/NO

2.6 Swelling: YES/NO

**3. OTHER ETIOLOGIES:** YES/NO *(specify if applicable)*
**3.1 Etiology:** ______________________
**3.2 Herniation:** YES/NO<br>**Type:** ○ transtentorial<br>○ subfalcine<br>○ Cerebellar tonsil
**3.2 Midline shift:** ____mm
**3.3 Basal cistern effacement:** YES/NO
**3.4 Swelling:** YES/NO
**3.5 Hydrocephalus:** YES/NO

COMMENTS
